# Role of Vascular Endothelial Growth Factor (VEGF) and VEGF-R Genotyping in Guiding the Metastatic Process in pT4a Resected Gastric Cancer Patients

**DOI:** 10.1371/journal.pone.0038192

**Published:** 2012-07-09

**Authors:** Mario Scartozzi, Cristian Loretelli, Eva Galizia, Alessandra Mandolesi, Mirco Pistelli, Alessandro Bittoni, Riccardo Giampieri, Luca Faloppi, Maristella Bianconi, Michela Del Prete, Francesca Bianchi, Laura Belvederesi, Italo Bearzi, Stefano Cascinu

**Affiliations:** 1 Clinica di Oncologia Medica, AO Ospedali Riuniti-Università Politecnica delle Marche, Ancona, Italy; 2 Centro Regionale di Genetica Oncologica, AO Ospedali Riuniti-Università Politecnica delle Marche, Ancona, Italy; 3 Oncologia Medica, Ospedale Profili, Fabriano, Italy; 4 Anatomia Patologica, AO Ospedali Riuniti-Università Politecnica delle Marche, Ancona, Italy; 5 Scuola di Specializzazione in Oncologia, Università Politecnica delle Marche, Ancona, Italy; University of Nebraska Medical Center, United States of America

## Abstract

In radically resected gastric cancer the possibility to predict the site of relapse could be clinically relevant for the selection of post-surgical management. We previously showed that specific tumour integrins genotypes are independently associated with either peritoneal or hematogenous metastases (ITGA and ITGV). Recently VEGF and VEGF-R polymorphisms have been demonstrated to potentially affect tumour angiogenesis and the metastatic process in gastric cancer. We then investigated the role of VEGFs and VEGF-R genotyping in determining either peritoneal carcinosis or hematogenous metastases in radically resected gastric cancer patients. Tumour genotyping for integrins (ITGA and ITGV) was also performed according to our previous findings. Genotyping for VEGF-A, VEGF-C, VEGFR-1,2,3 and ITGA and ITGV was carried out on pT4a radically resected gastric tumours recurring with either peritoneal-only carcinosis or hematogenous metastases. 101 patients fulfilled the inclusion criteria: 57 with peritoneal carcinomatosis only and 44 with hematogenous spread only. At multivariate analysis, intestinal histology and the AC genotype of rs699947 (VEGFA) showed to independently correlate with hematogenous metastases (p = 0.0008 and 0.008 respectively), whereas diffuse histology and the AA genotype of rs2269772 (ITGA) independently correlated with peritoneal-only diffusion (p = <0.0001 and 0.03 respectively). Our results seem to indicate that combining information from genotyping of rs699947 (VEGFA, AC)**,** rs2269772 (ITGA, AA) and tumour histology could allow clinicians to individuate gastric cancer at high risk for recurrence either with peritoneal or hematogenous metastases. The selection tool deriving from this analysis may allow an optimal use of the available treatment strategies in these patients.

## Introduction

The global outcome for radically resected gastric cancer patients remained substantially unchanged across the years in western Countries, with a 5-years survival rate ranging between 15% and 35% of all cases [1], [2]. Despite multiple efforts, most clinical trials investigating the role of post-operative treatments showed controversial results.

In western Countries only a modest survival benefit, has been observed for adjuvant chemotherapy mainly in pooled analyses and the beneficial effect of chemoradiotherapy seemed limited to patients receiving an inadequate clearance of the lymphatics [3], [4].

Peritoneal carcinosis may be the first site of relapse in approximately 40 to 50% of patients undergoing radical resection [5], [6], [7]. Based on these data a potential role for intraperitoneal chemotherapy in the prevention of peritoneal carcinomatosis has been hypothesized [6], [7]. However the containment of disease with a locoregional treatment may be inadequate in patients at high risk for hematogenous metastases and should be better reserved only to those likely to recur in the peritoneum without hematogenous diffusion. Unfortunately predictive factors for the site of recurrence and potentially able to suggest a personalized post-operative strategy are largely lacking. Although many clinical determinants have been analyzed in the past, only few of them proved to be effectively relevant in predicting the site of relapse. Clinical and pathological factors such as tumour serosal involvement, tumour histology (diffuse vs. intestinal), the extent of lymphadenectomy and the presence of tumour cell in peritoneal lavage during laparotomy have been indicated as predictive for the site of recurrence in gastric cancer [4], [8], [9].

In a previous analysis we found that Genotyping of rs2269772 (*ITGA*) and rs11902171 (*ITGV*) may represent a critical asset in the definition of high risk gastric cancer patients for peritoneal carcinosis [10].

The Vascular Endothelial Growth Factors (VEGF) superfamily has been identified to critically influence angiogenesis in solid tumours. Published data suggest that in presence of tumour angiogenesis-related factors distant metastases are more likely to occur [11], [12].

Interestingly it has been also demonstrated that the angiogenic phenotype may differ between intestinal-type and diffuse-type gastric cancer. In different analyses intestinal-type tumours seem in fact more biologically dependent on angiogenesis than diffuse-type tumours [13], [14], [15].

Single-nucleotide polymorphisms (SNPs) in the vascular endothelial growth factors (VEGF) and VEGF receptors (VEGF-R) genes have been correlated to tumour neoangiogenesis through different biological mechanisms. In patients with colorectal cancer specific SNPs in the VEGF-R2 have been shown to significantly influence microvessel density [16]. Specific SNPs in the VEGFA gene resulted able to determine response to chemotherapy through modulation of tumour blood vessels structure and function [17].

Tumour lymphangiogenesis is strictly dependent on the biological activity of the VEGFC and the VEGF-R3 [18], [19]. Interestingly lymphatic diffusion along with direct colonization of cancer cells also represent crucial, but biologically distinct, steps for peritoneal dissemination. Based on these assumptions we can then hypothesise that the altered expression of the VEGFC/VEGFR-3 pathway may influence peritoneal diffusion of cancer cells through the lymphatics [20]. On the other hand the VEGFC/VEGF-R3 pathway has been also demonstrated relevant for hematogenous cancer dissemination as well [21].

Predicting the location of recurrence amongst high risk individuals may allow for tailored adjuvant approaches. We therefore examined the role of tumour VEGFs and VEGF-Rs polymorphisms in determining either peritoneal carcinosis or hematogenous metastases in radically resected gastric cancer patients. Clinical data and SNPs results for tumour VEGFs, VEGF-Rs and integrins have been analysed with the aim to suggest a biologically-driven profile to be employed as a tool for the appropriate treatment selection in the appropriate patient.

## Methods

### Patients Selection

The patients study population was selected from a central database including patients affected by gastric cancer, operated in four different Institutions of our Region. Classification of the T, N and M-factors was made according to the numeric system introduced by the 7^th^ TNM.

Only patients who had recurred within 2 years, with either peritoneal-only carcinosis or hematogenous metastases after curative gastrectomy for a pT4a gastric adenocarcinoma, who did not receive adjuvant chemotherapy and whose tumour specimen was available were selected for tumour genotyping. Patients with either distant metastases at the time of diagnosis or presence of exfoliated tumour cells in peritoneal lavage obtained during laparotomy were excluded from analysis. We decided to restrict our analysis to patients with pT4a tumours as this group of patients is at higher risk for peritoneal diffusion and is often considered for locoregional treatment such as intraperitoneal chemotherapy. However a not negligible proportion of these patients does not recur locally and therefore a selection tool in this setting would be a particularly valuable asset for the treating physicians. Since most recurrences are registered within 2 years from diagnosis we chose to exclude patients recurring after this time period in order to obtain an homogenous population.

Follow-up of both groups (peritoneal-only or hematogenous metastases) occurred at three-months intervals. Follow-up consisted of physical examination, a complete blood count, chest radiography and US of the abdomen or CT scanning as clinically indicated. The site and date of first relapse and the date of death were recorded.

### Ethics Statement

This study was approved by Ethical committee AOU Ospedali Riuniti – Umberto I of our institution. All patients provided informed written consent.

### Tumour VEGF and VEGF-R Genotyping

VEGF and VEGF-R genotyping was performed on formalin-fixed paraffin-embedded tissue block (about 30 mg) of primary gastric cancer samples.

Paraffin wax was removed with xylene and the samples were washed twice with 100% ethanol. DNA was isolated from the deparaffinised tissue using the RecoverAll™ Total Nucleic Acid Isolation Kit for FFPE Tissues (Applied Biosystems, Foster City, CA, USA), according to the manufacturer’s instructions. DNA from each sample was then eluted in 120 µl of eluting solution.

Single nucleotide polymorphisms (SNPs) within each gene were selected using the Pupasuite software (http://pupasuite.bioinfo.cipf.es/index.jsf - version 2.0.0, bioinfo 2008), the CIPF Single Nucleotide Polymorphism database (dbSNP) generated by the National Center for Biotechnology Information (http://www.ncbi.nlm.nih.gov/SNP) and by review of the medical literature, using the following criteria:

1. the polymorphism had some degree of likelihood to alter the structure or the expression of the gene in a biologically relevant manner (i.e. affecting ese sequences, 3′ UTR or promoter region);
2. the minor allele frequency was above 10% (with the only exception of rs2305948, rs6877011 and rs307822);
3. the genetic polymorphism was established and well-documented.

Further considerations drove the selection of SNPs for our study. A correlation between the presence of a specific allele on a polymorphic site and the expression of the respective protein has been previously documented for VEGF [22], [23]. SNPs in regulatory sequences, such as introns and 5′ and 3′ UTRs, have been shown to alter mRNA stability [24], [25] processing efficiency [26], isoform expression [27], [28] and localization [29]. Moreover, regulatory motif sequences within the 3′ UTR of mRNAs have been shown to affect the stability of the messenger and/or its translational efficiency [28]. Thus, it can be argued that SNPs in these sequences may influence VEGF and VEGF-R gene expression. Also on these bases, we selected the SNPs known to affect VEGF and VEGF-R expression and those located in regulatory sequences, for which a putative role in protein regulation can be assumed.

Globally we assumed that selected SNPs had impact on protein expression and therefore on biological function.

Selected SNPs were as follows: six polymorphisms in the VEGFA gene (rs10434, G>A; rs2010963, G>C; rs25648, C>T; rs3025039, C>T; rs699947, A>C; rs833061, C>T), two in VEGFC (rs4604006, T>C; rs7664413, C>T), two in VEGF-R1 (FLT1) (rs664393, G>A; rs7993418, A>G), four in VEGF-R2 (KDR) (rs1870377, A>T; rs2071559, A>G; rs2305948, G>A; rs7667298, A>G) and three in VEGF-R3 (FLT4) (rs307805, A>G; rs6877011, C>G; rs307822, G>A). Chromosomal locations, positions and biological effects of investigated VEGF and VEGFR SNPs have been summarised in table 1.

**Table 1:** Chromosomal locations, positions and biological effects of investigated gene SNPs.

SNP ID	Gene	Chr	Chr. Position	Position in the gene/Effect	Codon exchange	aa. exchange
rs10434	VEGFA	6	43753212	3′UTR	–	–
rs2010963	VEGFA	6	43738350	5′UTR	–	–
rs25648	VEGFA	6	43738977	Syn; ESE	TCC ⇒ TCT	S [Ser] ⇒ S [Ser]
rs3025039	VEGFA	6	43752536	3′UTR	–	–
rs699947	VEGFA	6	43736389	promoter	–	–
rs833061	VEGFA	6	43737486	promoter	–	–
rs4604006	VEGFC	4	177608775	intronic	–	–
rs7664413	VEGFC	4	177608707	intronic	–	–
rs664393	FLT1	13	29071001	3′UTR	–	–
rs7993418	FLT1	13	28883061	Syn; ESE	TAC ⇒ TAT	Y [Tyr] ⇒ Y [Tyr]
rs1870377	KDR	4	55972974	Missense Subs.	CAA ⇒ CAT	Q [Gln] ⇒ H [His]
rs2071559	KDR	4	55992366	Init. Transcription	–	–
rs2305948	KDR	4	55979558	Missense Subs.	GTA ⇒ ATA	V [Val] ⇒ I [Ile]
rs7667298	KDR	4	55991731	5′UTR	–	–
rs307805	FLT4	5	180077487	Prom; TFBS	–	–
rs6877011	FLT4	5	180029471	3′UTR	–	–
rs307822	FLT4	5	180028717	3′UTR	–	–

syn: Synonymous substitution;

ESE: Exon Splicing Enhancer;

3′UTR: Untranslated Region 3′UTR;

5′UTR: Untranslated Region 5′UTR;

Prom: Promoter region;

TFBS: Predicted Trascription Factor Binding Site.

SNP genotyping was performed by TaqMan technology, using SNP genotyping products (Applied Biosystems). Polymerase chain reaction (PCR) was performed and genotypes were analysed on the 7300 Real-Time PCR System (Applied Biosystems) using an ABI Prism 7300 Sequence Detection System software (version 1.3.1, Applied Biosystems). Each reaction contained 0.2 µl of total genomic DNA. Genotyping was performed by laboratory personnel blinded to patient status, and a random 10% of the samples were repeated to validate genotyping procedures.

All SNPs genotyped had to present an overall call rates of ≥90% to be included in our analysis.

### Tumour Integrins Genotyping

Genotyping for tumour integrins independently associated with either peritoneal or hematogenous metastases in our previous analysis (A and G genotypes of rs2269772, *ITGA* and G and C genotypes of rs11902171, *ITGV*) was conducted as previously described [10].

### Statistical Analysis

Statistical analysis was performed with the MedCalc software version 10.4.8 for Windows. The association between categorical variables was estimated by Chi-square test. Logistic regression analysis was used to assess the independent role of variables resulted significant at univariate analysis.

Tested variables included sex (male vs. female), age (<65 yrs vs. ≥ 65 yrs), absence or presence of lymph node metastases (pN0 vs. pN+), type of lymphadenectomy (D1 vs. D2), tumour histology according to Lauren’s classification (intestinal vs. diffuse), lymphatic/blood vessels invasion (presence vs. absence), the clinical Center where surgery was performed, integrins genotyping (A and G genotypes of rs2269772, *ITGA* and G and C genotypes of rs11902171, *ITGV*) and each VEGF and VEGF-R polymorphism.

The ratio of the odds of the outcome (Odds ratio) in the 2 groups (peritoneal carcinosis only and hematogenous metastases) was calculated with a 95% confidence interval. A significant level of 0.05 was chosen to assess the statistical significance.

All polymorphisms were examined for deviation from Hardy-Weinberg equilibrium using the Powermarker v. 3.25 package (www.statgen.ncsu.edu/powermarker).

Linkage Disequilibrium (LD) analysis was also performed using the Powermarker v. 3.25 package (www.statgen.ncsu.edu/powermarker). LD was estimated using r2, with r2 = 1 indicating complete LD and r2 = 0 indicating absent LD.

## Results

### Hardy-Weinberg Equilibrium and Linkage Disequilibrium

The frequencies of the tested genotypes resulted comparable to those reported in Caucasians, with no significant deviation from the Hardy-Weinberg equilibrium.

Linkage disequilibrium was not observed for the tumour genotypes independently correlated with either peritoneal or hematogenous metastases.

### Patients Characteristics

One hundred and one patients were available for our analysis: 66 males and 35 females with a median age at diagnosis of 67 years (range 34–89). Follow-up data were available for all patients included. Follow-up consisted of physical examination, a complete blood count, chest radiography and US of the abdomen or CT/MRI scanning as clinically indicated. Only patients who exclusively developed either peritoneal carcinosis or hematogenous metastases during the entire course of their disease were considered.

All patients underwent radical surgery (R0) for pT4a gastric cancer, regional lymphnodes were negative for metastases (pN0) in 23 patients (23%). Most of the patients underwent a D2 lymphadenectomy (94 cases, 93%). Pathology report showed intestinal histology in 51 cases (51%) and diffuse histology in 50 patients (49%) (table 1). Fifty-seven patients (56%) developed peritoneal carcinosis-only tumour diffusion, whereas hematogenous metastases were diagnosed in the remaining 44 patients (44%). Clinicopathological variables of both groups (carcinosis-only and hematogenous metastases) were comparable with the exception of tumour histology. All major clinical and pathological characteristics are summarized in table 2.

**Table 2:** Patients characteristics.

	Patients		
	Peritoneal carcinosis	Hematogenous metastases	*p*
**N**	57	44	
**Age (range)**	63 (37–89)	61 (34–88)	
**Sex, n (%)**			n.s.
Males	33 (58)	33 (75)	
Females	24 (42)	11 (25)	
**Stage at diagnosis, n (%)**			n.s.
pT4a pN0	9 (16)	14 (32)	
pT4a pN+	48 (84)	30 (68)	
pN1	23 (40)	17 (39)	
pN2	18 (32)	12 (27)	
pN3	3 (5)	1 (2)	
**Tumour histology, n (%)**			<0.0001
Intestinal	11 (19)	40 (91)	
Diffuse	46 (81)	4 (9)	
**Lymphatic/blood vessels invasion, n (%)**			n.s.
Present	9 (16)	6 (14)	
Absent	48 (84)	38 (86)	
**Lymphadenectomy, n (%)**			n.s.
D1	5 (9)	3 (7)	
D2	52 (91)	41 (93)	

### Tumour VEGF and VEGF-R Genotyping

All SNPs genotyped presented an overall call rates of ≥90%.

Repeated samples for genotyping validation confirmed results obtained by previous analysis in all cases.

Gastric tumours showing the AA genotype of rs10434 (*VEGFA*), the AC genotype of rs699947 (VEGFA) and the GG genotype of rs7993418 (FLT1) resulted more prone to hematogenous metastases (peritoneal carcinosis vs. hematogenous metastases: 8% vs. 27%, p = 0.02; 30% vs. 59%, p = 0.006 and 2% vs. 16%, p = 0.02 respectively) (table 3). No correlation could be found between these tumour genotypes and peritoneal-only diffusion.

**Table 3:** Detailed results for tumour VEGF and VEGF-R polymorphisms associated with either peritoneal-only diffusion or hematogenous metastases at univariate analysis.

	rs10434 (VEGFA, G>A)			
	GG	GA	AA	ND
**Peritoneal Carcinosis** **n (%)**	16 (28)	30 (53)	5 (9)	6 (10)
**Hematogenous** **Metastases n (%)**	11 (25)	20 (46)	12 (27)	1 (2)
***p***	n.s.	n.s.	0.0282	
	**rs699947 (VEGFA, A>C)**			
	**AA**	**AC**	**CC**	**ND**
**Peritoneal Carcinosis** **n (%)**	9 (16)	17 (30)	26 (45)	5 (9)
**Hematogenous** **Metastases n (%)**	5 (11)	26 (59)	11 (25)	2 (5)
***p***	n.s.	0.006	n.s.	
	**rs7993418 (FLT1, A>G)**			
	**AA**	**AG**	**GG**	**ND**
**Peritoneal Carcinosis** **n (%)**	34 (60)	16 (28)	1 (2)	6 (10)
**Hematogenous** **Metastases n (%)**	21 (48)	13 (29)	7 (16)	3 (7)
***p***	n.s.	n.s.	0.0259	

ND = not done;

ns = not significant;

Analysis of the remaining VEGF and VEGF-R polymorphisms did not show any correlation with either peritoneal carcinosis or hematogenous metastases (table 4).

**Table 4:** Global results for tumour VEGF and VEGF-R polymorphisms and either peritoneal-only diffusion or hematogenous metastases at univariate analysis.

SNP ID	Gene	Peritoneal Carcinosis Or Hematogenous Metastases
rs10434	VEGFA	p = 0.0282
rs2010963	VEGFA	p = 0.8
rs25648	VEGFA	p = 0.5
rs3025039	VEGFA	p = 0.07
rs699947	VEGFA	p = 0.006
rs833061	VEGFA	p = 0.08
rs4604006	VEGFC	p = 0.4
rs7664413	VEGFC	p = 0.09
rs664393	FLT1	p = 0.6
rs7993418	FLT1	p = 0.0259
rs1870377	KDR	p = 0.06
rs2071559	KDR	p = 0.2
rs2305948	KDR	p = 0.09
rs7667298	KDR	p = 0.1
rs307805	FLT4	p = 0.5
rs6877011	FLT4	p = 0.07
rs307822	FLT4	p = 0.06

### Tumour Integrins Genotyping

Globally results for tumour integrins genotyping confirmed our previous findings.

Patients tumours with the GG genotype of rs2269772 (*ITGA*) resulted less prone to peritoneal carcinosis (peritoneal carcinosis vs. hematogenous metastases: 39% and 77% of patients respectively, p = 0.002), whereas the AA genotype of rs2269772 (*ITGA*) was more frequently associated to peritoneal carcinosis (peritoneal carcinosis vs. hematogenous metastases: 25% and 5% of patients respectively, p = 0.014). The CC genotype of rs11902171 (*ITGV*) was more frequently associated to peritoneal carcinosis (peritoneal carcinosis vs. hematogenous metastases: 21% and 5% of patients respectively, p = 0.016). On the contrary, patients with the GG genotype of rs11902171 in the *ITGV* gene developed peritoneal carcinosis less frequently than hematogenous metastases (peritoneal carcinosis vs. hematogenous metastases: 42% and 66% of patients respectively, p = 0.02).

### Other Clinical/pathological Factors

Among the other tested variables, tumour diffuse histology showed a correlation with peritoneal carcinosis (peritoneal carcinosis vs. hematogenous metastases: 81% and 9% of patients respectively, p<0.001), whereas tumour intestinal histology was linked to hematogenous metastases (peritoneal carcinosis vs. hematogenous metastases: 19% and 91% of patients respectively, p<0.001) (table 2).

### Multivariate Analysis

At multivariate analysis, intestinal histology and the AC genotype of rs699947 (VEGFA) showed to independently correlate with hematogenous metastases, whereas diffuse histology and the AA genotype of rs2269772 (ITGA3) independently correlated with peritoneal-only diffusion (p = 0.001). Odds ratio results for these latter factors confirmed their role in guiding tumour cells through the metastatic process toward the peritoneum or hematogenous sites (table 5).

**Table 5:** Odds ratio results for variables resulted independently correlated with either peritoneal or hematogenous metastases at multivariate analysis.

	OR for peritoneal carcinosis	*p*	95% CI
**diffuse histology**	4.6	<0.0001	2.2–12.3
**rs2269772 (ITGA, AA)**	12.2	0.03	1.8–29.9
	**OR for hematogenous metastases**	***p***	**95% CI**
**Intestinal histology**	4.2	0.0008	2–10.2
**rs699947 (VEGFA, AC)**	8.08	0.008	1.7–37.5

## Discussion

The introduction of multiple therapeutic options for the post-surgical management of radically resected gastric cancer patients has opened the question of optimal patients selection. Unfortunately a critical limiting factor for the potential of these treatment choices is represented by the lacking of clinical and biological factors able to predict the site of recurrence. The containment of disease with a locoregional treatment such as intraperitoneal chemotherapy may be in fact inadequate in patients at high risk for hematogenous metastases and should be better reserved to patients most likely to recur with peritoneal-only diffusion. On the other hand in patients more likely to recur with hematogenous metastases adjuvant chemotherapy may represent a more effective strategy.

In our experience the rs699947 (VEGFA, AC) polymorphism is independently related to a higher risk of hematogenous metastasis with an OR 8.08 (95% CI 1.7–37.5, p = 0.008). SNPs in the promoter region of the VEGF gene have been already demonstrated to affect patients outcome in colorectal, breast and gastric cancer [17], [30], [31]. The biological interference on VEGF gene function caused by polymorphic variants in the promoter region is in fact likely to determine an altered expression of the gene. This may both increase the tumour-related pro-angiogenesis potential and ultimately guide the metastatic process through the formation of new blood vessels [16], [17], [30], [31]. However no data are available about the role of VEGFs and VEGF-Rs genotyping in determining the metastatic process in radically resected gastric cancer. Most studies investigated only few SNPs potentially involved in tumour angiogenesis, whereas in the present study multiple SNPs in the VEGFs genes and receptors have been globally analysed along with other known determinants.

As previously described the rs2269772 (ITGA, AA) genotype of tumour integrins confirmed to be independently relevant in determining peritoneal diffusion of gastric cancer cells [10). This finding deriving from a multivariate analysis including tumour-angiogenesis related factors strongly suggests that this specific tumour integrin polymorphism should be considered in future studies. On the contrary our analysis could not confirm the role of tumour integrins in determining hematogenous diffusion. We know from previously published analyses that upregulation of integrins sub-classes could enhance neoplastic cells motility and VEGF expression [32], . Integrins may consequently loose their function in determining hematogenous tumour diffusion when angiogenesis-related factors are already activated.

Tumour histology resulted a further factor independently able to identify the gastric cancer potential to determine either peritoneal or hematogenous metastases. Nonetheless tumour histology alone does not seem sufficient to accurately predict the metastatic sites. In our experience, similarly to previous reports, a relevant proportion of patients developed peritoneal carcinosis or hematogenous metastases independently from tumour histology. Similar considerations could be applied to the presence of tumour serosal involvement and/or exfoliated cancer cells in peritoneal lavage. Not all gastric tumours with serosal infiltration will eventually develop carcinosis and a negative peritoneal lavage unfortunately does not exclude a future peritoneal diffusion [8], [9]. We demonstrated that genotyping of rs699947 (VEGFA, AC) and rs2269772 (ITGA, AA) have an independent role in determining the metastatic site of gastric cancer cells even when tumour histology is included in the analysis. Our results seem then to indicate that combining information from genotyping of rs699947, rs2269772 and tumour histology could allow clinicians to individuate gastric cancer at high risk for recurrence either with peritoneal or with hematogenous metastases among patients undergoing apparent radical surgery for pT4a cancer without exfoliated cancer cells in peritoneal lavage. In our study DNA genotyping was performed only on tumour DNA and this may represent a limitation for data interpretation.

Globally we believe that genotyping for specific VEGFA and integrin genes may represent a critical asset in the definition of the metastatic diffusion for radically resected gastric cancer patients. Radically resected gastric cancer patients at high risk for peritoneal recurrence may be optimal candidates for intraperitoneal therapy, whereas if the risk of hematogenous metastases is predominant systemic chemotherapy may represent a better option. The selection tool deriving from this analysis may then allow an optimal use of the available treatment strategies and should represent a possible stratification factor in future studies in these patients.
